# Use of Robotic Surgery in Plastic and Reconstructive Surgery: A Narrative Review

**DOI:** 10.3390/biomimetics10020097

**Published:** 2025-02-09

**Authors:** Jennifer Novo, Ishith Seth, Yi Mon, Akshay Soni, Olivia Elkington, Gianluca Marcaccini, Warren M. Rozen

**Affiliations:** 1 Faculty of Medicine and Surgery, University of Notre Dame, Sydney, NSW 2008, Australia; jenny.novo@gmail.com; 2 Department of Plastic and Reconstructive Surgery, Peninsula Health, Melbourne, VIC 3199, Australiaakshaysoni022@gmail.com (A.S.);; 3 Faculty of Medicine and Surgery, Monash University, Melbourne, VIC 3004, Australia

**Keywords:** robotic surgery, plastic surgery, reconstructive surgery, biomimetics, surgical innovations

## Abstract

Background/Objectives: Robotic systems offer enhanced precision, dexterity, and visualization, which are essential in addressing the complex nature of plastic surgery procedures. Despite widespread adoption in other surgical specialties, such as urology and gynecology, their application in plastic surgery remains underexplored. This review examines the use of robotic systems in plastic and reconstructive surgery with a focus on clinical outcomes. Methods: A literature search was conducted using PubMed, Embase, Scopus, and Web of Science. Search terms included (“robotic surgery” OR “surgical robots”) AND (“plastic surgery” OR “reconstructive surgery”). Studies on clinical outcomes and biomimetic innovations published between 1980 and 2024 were included, while non-English, cadaver-based, and animal studies were excluded. Data were systematically extracted using Covidence and analyzed. Results: Twenty-nine studies were identified that evaluated the clinical outcomes of robotics in areas including breast reconstruction, microsurgery, and craniofacial procedures. Robotic systems like the Da Vinci and Symani platforms offer motion scaling, tremor elimination, and enhanced depth perception. In nipple-sparing mastectomies, they reduced skin necrosis rates from 8% to 2%, while in DIEP flap reconstruction, they enabled smaller fascial incisions (2.67 ± 1.13 cm vs. 8.14 ± 1.69 cm) and faster recovery with fewer complications. In microsurgery, they achieved 100% patency for vessels under 0.3 mm and a 25.2% limb volume reduction in lymphedema patients in 3 months. Conclusions: Robotic systems show significant promise, particularly in procedures such as nipple-sparing mastectomies, and have the potential to overcome challenges including surgeon fatigue. However, challenges such as longer operating times, high costs, and limited haptic feedback remain barriers to their adoption.

## 1. Introduction

Biomimetics is the interdisciplinary practice of emulating nature and its processes to address human challenges, providing evolutionary and efficient solutions to conventional methods [1]. Biomimetics has played a transformative role in medicine, especially in surgical practices. Examples include snake-like endoscopes that allow for navigating intricate parts of the body and robotic arms inspired by the flexibility of cephalopod limbs and the ability to bend in all directions [2,3]. These advancements have significantly contributed to minimally invasive surgeries by improving surgical precision, reducing complications, and shortening patient recovery times. For instance, flexible robotic systems have allowed surgeons to perform procedures with enhanced control in anatomically challenging regions, such as the pelvis or cranial cavity [2,3].

Of these advancements, the plastic and reconstructive surgery field has benefited from biomimetic principles. Procedures in this field, such as microsurgery, breast reconstruction, and craniofacial surgery, demand extraordinary precision, skill, and versatility, qualities that biomimetic robotic systems can uniquely provide [3,4]. Robotic platforms like Da Vinci and Symani have incorporated features such as articulated robotic arms that mimic human movement and allow precise motion scaling [4,5]. These innovations offer unmatched precision and visualization, enabling surgeons to perform intricate procedures with significantly improved outcomes by reducing unnecessary movements that may damage nearby intricate structures [4,5].

Integrating biomimetic innovations in robotic surgery has allowed surgeons to push the boundaries of their surgical capabilities. These platforms address physical limitations inherent in manual techniques, such as tremors, fatigue, and restricted access to deep or complex anatomical sites, all of which can compromise surgical outcomes. In plastic and reconstructive surgery, robotic platforms have allowed breakthroughs such as robotic-assisted nipple-sparing mastectomies (RANSM), which provide adequate exposure while preserving critical vasculature and minimizing tissue damage [4,6]. Additionally, minimally invasive approaches have reduced abdominal wall morbidity and improved the precision of pedicle dissection in robotic deep inferior epigastric perforator (DIEP) flap reconstruction [4,7]. Robotic systems have also been instrumental in supermicrosurgical lymphaticovenular anastomoses for lymphedema treatment, allowing surgeons to operate on vessels smaller than 1 mm in diameter through enhanced magnification and motion control [4].

Despite these advancements, plastic and reconstructive surgery continues to face significant challenges. Critical areas for improvement include optimizing tissue manipulation, achieving superior microsurgical precision, and reducing surgeon fatigue during lengthy procedures. These challenges arise from the inherent complexity of reconstructive surgeries, which often involve intricate anatomical structures and demand sustained precision. Even when refined, manual techniques are prone to limitations, such as tremors, restricted dexterity, and physical strain on surgeons [3,7]. Biomimetic-inspired surgical technology addresses these issues by enhancing precision, providing meticulous control over tissue manipulation, and integrating innovations like artificial intelligence and tactile feedback [8]. These systems provide real-time sensory information to surgeons, augmenting their ability to sense tissue texture and resistance, ultimately improving outcomes [8]. Robotic platforms such as the Da Vinci system also reduce physical strain through their ergonomic design, allowing surgeons to maintain focus over extended periods [7]. By reducing physical and cognitive burdens, biomimetic-inspired robotics enhance clinical outcomes and surgeon well-being, making them essential tools for advancing plastic and reconstructive surgery [4,7]. This narrative review aims to evaluate the current applications of robotic surgery in plastic and reconstructive procedures while exploring how biomimetics has driven innovation over the last four decades to advance surgical practice.

## 2. Materials and Methods

This narrative review synthesizes evidence from published literature to explore the integration of biomimetics and robotic surgery in the plastic and reconstructive field. Two authors (JN and YM) searched databases, including PubMed, Embase, Scopus, and Web of Science, covering articles published from 1 January 1980 to 30 November 2024 with discrepancies reviewed by a third author. The search used specific keywords that were informed by a preliminary scoping review to identify commonly used terms in the field. Search terms included “robotic surgery” or “surgical robots” in combination with “plastic surgery” or “reconstructive surgery” to target studies directly related to the scope of this review. Boolean operators such as (“robotic surgery” OR “surgical robots”) AND (“plastic surgery” OR “reconstructive surgery”) AND (“biomimetics” OR “biomimicry” OR “nature-inspired”). Additional search terms, including “Symani robotic” and “Da Vinci robotic system”, were included to ensure a comprehensive search. Search terms were adapted for each database where required. The references of all included studies were thoroughly reviewed to identify additional relevant literature.

### Inclusion and Exclusion Criteria

The review included peer-reviewed articles, such as cohort studies, randomized controlled trials, prospective studies, and case series, available in English. Studies were included if published between 1980 and 2024 and focused on the use of robotic systems in plastic and reconstructive surgery, biomimetic principles, or features in robotic design.

Studies were excluded if they were non-English, involved cadavers or animal studies, or were unrelated to plastic and reconstructive surgery, reviews, case reports, or conference papers. Inclusion criteria were studies that investigated the use of surgical robots in the plastic and reconstructive surgical field.

Data extraction was performed using Covidence (2024), a systematic review management tool, to assist with the screening and study analysis process. Extracted data included study type (e.g., case series, clinical trial), robotic system or device used (e.g., Da Vinci, Symani), biomimetic features (e.g., tactile sensors, flexible joints), surgical outcomes (e.g., precision, patient recovery), and limitations or challenges discussed in the studies. Extracted data were organized into tables to allow for systematic comparison and analysis of robotic systems, their biomimetic designs, and their clinical impact. This structured approach provided a comprehensive understanding of the integration of biomimetics in robotic surgery and its relevance to plastic and reconstructive procedures.

## 3. Results

### 3.1. Literature Search and Study Characteristics

The database searches identified 1397 studies. After removing duplications and excluded studies, 29 studies met the inclusion criteria and were included in this narrative review.

The included studies were published between 1 January 1980 and 30 November 2024. They examined the use of robotic surgery in plastic and reconstructive surgery across their primary domains: microsurgery, breast reconstruction surgery, and facial reconstruction surgery. Microsurgery was the most extensively studied, comprising 10 studies focusing on nerve repair, vascular anastomosis, and hand microsurgery. Eight studies examined robotic applications in breast reconstruction surgery, encompassing DIEP flap harvesting, latissimus dorsi muscle flap harvesting, and NSM. The remaining nine studies focused on head and neck reconstruction, craniofacial contouring, robotic-assisted upper face rejuvenation, and hair transplantation surgery. The included studies utilized robotic systems such as the Symani Surgical System, Da Vinci platforms, and ARTAS robotic systems. Several studies also highlighted the role of robotic systems in facilitating innovative combinations of reconstructive techniques, such as simultaneous robotic-assisted flap harvesting and microsurgical anastomosis, particularly in complex reconstructions like head and neck cancer defects or extensive burn scars [9]. A summary of the characteristics of these studies can be found in Table 1.

### 3.2. Current Applications of Robotic Surgery

Robotic systems have demonstrated transformative potential across various plastic and reconstructive surgery areas. In microsurgery, studies predominantly used the Symani Surgical System, which allowed for intricate procedures such as lymphaticovenous anastomoses (LVA) and nerve repair. This system provided motion scaling, tremor elimination, seven degrees of freedom, and enhanced visualization, effectively handling vessels smaller than 0.3 mm [32]. Similarly, robotic-assisted microsurgery has shown increasing utility in hand surgery, where the Symani Surgical System has been used for precise vessel manipulation and demonstrated favorable outcomes in traumatic replantation cases [24]. Advanced use cases have also emerged in peripheral nerve reconstruction, where robotic platforms assist in simultaneous multiple nerve coaptations and ensure optimal tension and alignment during repair, significantly improving functional outcomes [34].

Robotic systems were widely applied in breast reconstruction procedures, including DIEP flap harvesting, NSM, and latissimus dorsi muscle flap procedures. Wittesaele et al. [20] reported that robotic-assisted DIEP flap procedures allowed for smaller fascial incisions and enhanced intraoperative precision in pedicle dissection, although operative times were longer than conventional methods. Jung et al. [33] noted that RANSM using the Da Vinci Xi system demonstrated a learning curve, significantly reducing total operative time after the 21st procedure. The mean total operative times decreased from 378.1 min in the early phase to 334.2 min in the late phase, indicating improved efficiency over time [33]. Furthermore, robotic platforms have allowed for enhanced preservation of abdominal wall integrity during flap harvesting, reducing postoperative hernia rates and associated complications [22,35,36].

In head and face procedures, robotic systems were used in craniofacial contouring, aesthetic surgery, and hair transplant surgery. In robotic-assisted upper face rejuvenation, the Da Vinci system demonstrated improved surgical precision and ergonomic advantages for surgeons [37]. The ARTAS robotic system was used for robotic hair transplantation, benefiting from intelligent algorithms to optimize hair graft placement, avoiding injury to existing hairs, and improving patient satisfaction and surgeon efficiency [18].

### 3.3. Biomimetic Influences in Robotic Surgery

The robotic surgical systems incorporated various advanced biomimetic principles by replicating natural human movements and sensory capabilities, enhancing their functionality, especially for complex procedures. The Symani Surgical System incorporates features and biomimetic technologies such as motion scaling, tremor elimination, and NanoWrists with up to 7 degrees of freedom, which mimic the dexterity and steadiness of human hands. These features enhance precision and the delicate handling of tissues in microsurgery and nerve repair, even in deep anatomical planes [14,23,27,38]. These features were crucial in reducing surgeon fatigue and improving precision during supermicrosurgical lymphatic procedures [21]. Moreover, motion scaling of up to 20-fold and tremor filtering inspired by natural dexterity enabled safer handling of submillimeter vessels, which would be impossible otherwise [25,27].

Similarly, the Da Vinci platforms emulate human wrist movement with articulated robotic arms and also provide high-definition 3D imaging that replicates binocular vision, enhancing depth perception and precision in anatomically challenging surgical fields [30,37].

In addition, the ARTAS robotic system utilizes intelligent, nature-inspired algorithms to optimize graft placement in hair restoration surgery, achieving outcomes that resemble the efficiency of natural patterns [18]. The ARTAS system also uses stereoscopic imaging, mimicking human visual perception and enhancing speed and consistency in graft placement while reducing surgeon fatigue [18]. The biomimetic principles of the ARTAS system have contributed to its reliability and precision in achieving aesthetically satisfactory outcomes, as reflected in patient satisfaction scores as high as 4.13 out of 5 [18]. Surgeons have also expressed favorable evaluations of the system, with satisfaction scores averaging 4.10 out of 5, irrespective of operator experience, highlighting its usability across various clinical settings [18].

### 3.4. Clinical Outcomes

This review highlights significant improvements in surgical precision, reduced complication rates, enhanced patient satisfaction, and improved surgeon comfort and efficacy. In NSM, robot systems demonstrated reduced postoperative complication rates and improved patient aesthetic outcomes [13,30,35]. For example, studies consistently showed significantly lower skin necrosis rates in RANSM cases compared to conventional methods, with Kim et al. [30] reporting a reduction from 8.0% to 2.0% (*p* = 0.05), and Lai et al. [13] further supporting these findings. Patient-reported outcomes, particularly in sexual well-being scores, were also significantly higher (BREAST-Q: 50.7 vs 38.6, *p* = 0.001) [14,30]. Similarly, in a study by Ahn et al. [14], high patient psychosocial well-being scores following RANSM were noted compared to conventional methods, and invisible scars in the frontal view contributed to greater patient satisfaction.

Additionally, robotic-assisted breast surgery was associated with significantly reduced postoperative pain compared to conventional techniques [35,36]. Lee et al. [35] reported a mean pain intensity reduction from 3.1 +/− 1.1 in the conventional group to 2.3 +/− 0.9 in the robotic group during the first 24 h postoperatively (*p* = 0.0001). This finding is complemented by Bishop et al. [36], who also observed reduced patient-reported pain on the robotic side in bilateral DIEP flap cases.

In microsurgery, robotic platforms such as the Symani Surgical System have facilitated complex reconstructions, including multi-vessel anastomoses in free tissue transfers for extensive soft tissue defects. Dastagir et al. [24] demonstrated the efficacy of this robotic system in emergency hand trauma care, achieving 100% anastomotic patency with no postoperative complications. Further, Aman et al. [23] noted that robotic-assisted peripheral nerve surgeries provided precise coaptations with an average time per stitch of 4.5 min. Moreover, the Symani Surgical System has consistently improved surgical outcomes across multiple studies. Barbon et al. [38], for instance, reported that operative time during robotic-assisted LVA decreased from 23.9 min to 16.3 min over successive cases. Similarly, Weinzierl et al. [21] reported a 25.2% reduction in limb volume 3 months following robotic LVAs, with no reported complications at follow-up. Collectively, these findings reflect increased efficiency as more cases are performed, strengthening the clinical reliability and usability of robotic microsurgery [21,38].

In aesthetic and craniofacial applications, robotic platforms provided superior control over soft tissues, leading to natural results with minimal postoperative swelling [37]. The enhanced visualization and articulation of the robotic arms have been beneficial in subperiosteal and subtemporal dissection and contouring, reducing intraoperative strain on surgeons and improving patient outcomes [37]. Furthermore, force feedback mechanisms integrated into robotic systems have enhanced tactile sensitivity during delicate procedures such as genioplasty, allowing for greater accuracy in osteotomies and minimizing the need for revisions [17]. Lai et al. [9] demonstrated similar benefits in robot-assisted head and neck reconstructions, reporting 100% flap survival in 17 cases following precise vascular suturing of structures as small as 2.1 +/− 0.8 mm with minimal postoperative complications.

## 4. Discussion

Robots are increasingly being incorporated into several different surgical subspecialties, such as gynecology and urology, and their use can be particularly advantageous in the field of plastic and reconstructive surgery as well. Robotic platforms commonly used in plastic and reconstructive surgery make it possible to operate with extreme precision thanks to features like motion scaling and tremor elimination [39,40,41]. This translates into minimal and highly accurate surgical maneuvers, essential when working on delicate anatomical structures or in areas that are difficult to access. Robotic instruments, equipped with articulated arms and ergonomic control systems, help reduce the procedure’s invasiveness, causing less tissue trauma and faster patient recovery. Furthermore, the miniaturization of some components decreases the footprint in the operating room, improving the overall efficiency of the surgical team. This is well demonstrated in studies such as Wittesaele et al. [20], which reported that robotic-assisted DIEP flap harvests preserved fascial integrity and eliminated the need for postoperative drains by days four and five. Ahn et al. [14] further highlighted that robotic-assisted NSM achieved high patient satisfaction, largely due to minimal scarring and precise tissue handling made possible by advanced robotic systems.

Robotic-assisted surgery has expanded the range of possible plastic and reconstructive surgery procedures. In particular, it has proven crucial for microsurgery and supermicrosurgery procedures, such as complex vascular reconstructions and lymphedema treatments [41]. The ability to manipulate very small structures with maximum stability and enhanced visualization represents a decisive advantage in areas like breast reconstruction, craniofacial surgery, and facial aesthetic procedures. Moreover, the high dexterity offered by robotic systems makes it easier to access complex anatomical regions, reducing the risk of accidental damage to critical structures like nerves and major blood vessels [40,42]. This was reflected in Lai et al. [9], where robotic systems enabled 100% vascular patency in microvascular anastomoses during head and neck free flap reconstructions. Similarly, Wong et al. [31] demonstrated 100% DIEP flap survival in the da Vinci Robot group, further highlighting the safety of robotic systems in accessing complex anatomical sites through small incisions.

Biomimetics provides the theoretical and practical foundation for developing solutions that mimic biological systems to optimize the efficiency and versatility of surgical instruments. Observing the features of animals with remarkable flexibility, such as cephalopods, has influenced the design of robotic arms capable of fluid, multi-directional movements. Similarly, the anatomy and physiology of the human hand have inspired robotic joints that replicate pronation and supination, offering more intuitive control in precision maneuvers, such as microvascular sutures [42]. This biomimetic perspective makes combining mechanical robustness and delicate handling possible, key factors for achieving optimal plastic and reconstructive surgery results.

This can be seen especially in breast and reconstruction surgeries where the da Vinci Surgical systems utilized the mimicry of human hand and wrist movements to enhance precision and control, minimize scarring, and improve aesthetic outcomes [13,15,16,19,20,31,35]. It must also be noted that the flexibility of robotic arms may lead to collisions in the operating theatre, as observed by Chung et al. [10], highlighting the future need for a larger theatre room with robots. The application of biomimetic principles is not limited to the mechanical architecture of robots but also includes research on advanced materials and control algorithms. Coatings that imitate biological surfaces are being developed to increase selective adhesion or reduce friction. At the same time, algorithms inspired by natural learning and adaptation processes help refine instrument positioning and interaction with surrounding tissues, preserving their integrity. This is crucial in fields where stability and tactile sensitivity play a decisive role, such as free flap dissection or the suturing of microscopic vessels.

Future advances in innovation could involve the transition from bulky robots such as the Da Vinci and Symani surgical systems to “soft robots”, which have greater flexibility and adaptability to their surroundings [43]. “Soft robots” made of flexible materials are inspired by organisms like jellyfish or starfish, which exhibit remarkable adaptability to their surroundings. This technology offers safer handling of delicate tissues, as the pressure exerted is distributed and constant, reducing the risk of injury. The ability to deform and conform to anatomical surfaces may revolutionize reconstructive surgery, mainly when operating in irregular or hard-to-reach areas.

The highly effective adhesion observed in geckos, even on smooth surfaces, is a reference model for developing new sensors and anchoring systems [44]. In plastic and reconstructive surgery, such innovation would improve the grasp on slippery or fragile tissues, lowering the risk of tearing or bleeding. Adhesive microstructures and next-generation tactile sensors could enable safer and more precise handling, especially in vascular suturing, flap grafts, or composite tissue transfers.

As artificial intelligence advances, its role in robotic surgery will become increasingly significant, enabling optimized preoperative planning, virtual simulations, and even real-time evaluation [45,46,47]. Machine learning systems can recognize relevant tissues and anatomical structures, highlight potential risk areas, and offer advice on dissection or suturing methods. Operationally, these better balances speed and delicate execution, minimizing errors and complications, particularly in long or highly complex interventions [45].

The introduction of augmented and virtual reality systems is another evolutionary step for robotic-assisted surgery. By combining diagnostic imaging, such as CT scans or MRIs, with 3D simulation tools, surgeons can visualize the procedure in detail and identify potential anatomical challenges in advance. During the operation, augmented reality can overlay critical information directly onto the surgical field, facilitating the localization of vital structures and making tissue resection, dissection, and reconstruction maneuvers safer. This synergy of technologies also has a notable educational advantage, allowing residents and junior surgeons to learn more interactively. Lin et al. [17] highlighted that augmented reality systems improved the precision of preoperative planning and intraoperative execution in robotic-assisted genioplasty, demonstrating the potential of these technologies in refining surgical outcomes.

Combining these elements—from enhanced precision and minimally invasive procedures to biomimetic inspiration and onward to future possibilities in miniaturization and artificial intelligence—makes robotic-assisted surgery in plastic and reconstructive surgery a rapidly expanding field. The ultimate goal is to improve both the quality of the procedures and the patient experience, reducing risks and maximizing recovery speed. Progress in this area will depend on the ability of multiple disciplines (medicine, engineering, and biology) to collaborate in an interdisciplinary manner, leading to sustainable technological developments and an increasingly positive impact on the standard of care.

While robotic-assisted procedures demonstrated several advantages, they were not without challenges. Almost half of the included studies experienced longer operative times than conventional techniques, as noted in an earlier literature review [30]. Operative times were often prolonged due to the robotic system’s setup, docking, and intraoperative adjustments. While an initial learning phase is required for surgeons to gain proficiency with robotic techniques, studies demonstrate that operating times improve significantly with experience [14,18,24]. For instance, Wittesaele et al. [20] reported a mean operative time of 479 min for robotic-assisted DIEP flap harvests, while Kim et al. [30] found that robotic-assisted implant-based breast reconstruction required 252 +/− 38.5 min compared to 183 +/− 46.5 min using conventional methods. Lai et al. [13], Ahn et al. [14], and Jung et al. [33] also demonstrated that robotic NSM had prolonged total operation times, particularly during the early phase as surgeons gained experienced.

Integrating advanced materials and algorithms into robotic systems represents a pivotal step in addressing current limitations and expanding their applications in plastic and reconstructive surgery. In the future, emerging biomimetic materials, such as gecko-inspired adhesives and flexible polymers, may be used to enhance robotic precision and adaptability [43,44]. For instance, these gecko-inspired adhesives, which mimic the microfibrillar structures of gecko feet, could provide improved grip on smooth or delicate tissues without causing damage [44]. Similarly, flexible polymers and soft robotic materials might allow for more conformal interactions with complex anatomical surfaces, potentially minimizing the risk of tissue trauma during intricate maneuvers and expanding the range of feasible surgical techniques [43].

In parallel, significant advancements in algorithm development are transforming robotic capabilities. Artificial intelligence driven systems are being incorporated to optimize preoperative planning, intraoperative precision, and postoperative outcomes. Machine learning models are increasingly utilized to provide real-time analysis of anatomical structures, enhancing motion control and predicting potential complications. Intelligent feedback algorithms are also being developed to simulate tactile sensations, compensating for the lack of haptic feedback in many robotic systems. Additionally, AI-enhanced vision systems facilitate tissue recognition and dissection planning, improving precision and efficiency in microsurgical procedures.

Furthermore, high costs associated with robotic systems, their size and maintenance, and spatial constraints in operating rooms were consistently noted across studies and may hinder the adoption of robotic surgery; these limitations are comprehensively outlined in the table of study characteristics (Table 1). Additional advantages and disadvantages of different robotic systems can be seen in Table 2. Future studies with a cost-effective analysis would be beneficial in better assessing the actual costs of robotic surgery. Researchers should focus on compact, more affordable, and easily transportable systems, ideal for settings with limited resources or mid-sized healthcare facilities. This strategy could extend the benefits of robotic-assisted surgery to many patients, improving access to complex procedures even in less privileged geographical areas.

In addition to some of these challenges, several studies highlighted that the lack of tactile feedback in their robotic systems was a significant limitation, requiring surgeons to rely solely on visual cues during procedures. In the works of Farr et al. [28], Barbon et al. [38], and Lai et al. [9], the absence of haptic feedback posed significant challenges during complex tissue manipulations and microsurgical tasks. These limitations collectively represent the current hurdles in the broader use of robotic systems in plastic and reconstructive surgery.

## 5. Conclusions

The transformative potential of biomimetics in robotic surgery is reshaping the field of plastic and reconstructive surgery, promising unprecedented precision, versatility, and patient outcomes. By mimicking biological systems, these innovations enhance the functionality and adaptability of robotic systems, facilitating safer and more effective interventions in delicate and complex anatomical regions. Innovations such as soft robotics and gecko-inspired adhesives remain in early developmental stages and have yet to transition into clinical applications. While these technologies show promise for enhancing precision and safety in surgical robotics, further research is required to adapt and validate them for practical use in operating room environments. These technologies are in the early stages of development, with many yet to transition from experimental design to clinical applicability, particularly in reconstructive and microsurgical procedures.

However, realizing the full potential of this technology hinges on interdisciplinary collaboration. Integrating expertise from medicine, engineering, biology, and computer science is essential for addressing current limitations, such as high costs, lack of haptic feedback, and additional theatre space. Future research must prioritize accessibility, scalability, and usability, ensuring these cutting-edge tools benefit patients globally, including those in resource-limited settings. Through such collaborative efforts, robotic surgery in plastics and reconstructive surgery is set to redefine surgical standards, elevating precision, enhancing patient satisfaction, and accelerating recovery, ushering in a transformative era for the field.

## Figures and Tables

**Table 1:** Characteristics of included studies.

Author and Year	Study Design	Type of Plastic Surgery	Robotic System Used	Biomimetic Features	Surgical Outcomes	Limitations and Challenges
Chung et al., 2015 [10]	Non-randomized experimental study	Breast reconstruction: RA latissimus dorsi muscle flap	da Vinci Surgical System	Flexible joints; motion scaling; tremor control	High patient satisfaction rates for aesthetics (mean scores: 9.2 to 9.9/10) and no donor site complications; improved aesthetics with hidden scars; docking time and operating time improved with experience	Difficulty dissecting anterior border due to simultaneous robot arm overlap and confined working space; large workspace required; increased time due to retractor adjustments; steep learning curve
Nadjmi 2016 [11]	Case series	Head and neck: cleft palate reconstruction	da Vinci Surgical System	3D endoscopic vision; tremor filtration; EndoWrist simulating hand-like movements	Improved dexterity and precision in intraoral suturing; shortened hospital stays (1 vs. 2.4 ± 1.3 days); normal swallowing restored on surgery day	Lack of tactile feedback; longer operative times (122 ± 8 min vs. 87 ± 6 min); high costs; steep learning curve
Arora et al., 2018 [12]	Case series	Head and neck: robotic resection of tumor with free flap reconstruction	da Vinci Si Surgical System	Flexible robotic arms; high-definition 3D binocular vision; tremor elimination	100% flap survival; satisfactory functional and aesthetic outcomes; no postoperative complications; reduced surgical morbidity	Difficulty reconstructing flaps in narrow areas; limited access to recipient vessels; steep learning curve; longer docking/setup times
Lai et al., 2019 [13]	Case series	Breast reconstruction: RANSM with breast reconstruction	da Vinci Si Surgical Robot	3D imaging providing enhanced spatial precision; flexible robotic arms for fine dissection	87% of patients were graded as “excellent” for cosmetic outcomes; no total nipple-areolar complex necrosis was observed	Longer operative times; high costs
Lai et al., 2019 [9]	Case series	Head and neck: free flap reconstruction for oropharyngeal cancer	da Vinci Surgical System	High-definition 3D visualization; tremor elimination	100% flap survival rate; precise suturing of vessels as small as 2.1 mm; no major complications reported	Lack of tactile feedback; long setup and operating times; high costs
Ahn et al., 2019 [14]	Case series	Breast reconstruction: RANSM with immediate breast reconstruction	da Vinci Xi Surgical System	Flexible robotic arms; 3D magnified imaging	High patient satisfaction scores (BREAST-Q); invisible scars in most cases; no major complications reported	High costs and initial long operative times; securing workspace was challenging
Moon et al., 2020 [15]	Case series	Chest reconstruction: RA latissimus dorsi muscle flap surgery for Poland Syndrome	da Vinci Surgical System	3D camera for a magnified view; robotic arms mimicking hand movements	High patient satisfaction for aesthetics and chest symmetry; inconspicuous scars achieved; no serious complications or flap loss reported	Lack of control group; high cost associated with robotic system purchase and maintenance; insufficient follow-up; prolonged operative time; potential bias in scar assessment
Jeon et al., 2021 [16]	Cohort study	Breast reconstruction: RA immediate prosthetic breast reconstruction	da Vinci Xi Surgical System	High-definition 3D imaging; articulated and flexible arms with precision motion; tremor reduction	Mean operative time: 194.7 min (oncology team), 80.8 min (plastic surgery team); minor complications (6% seroma, 6% superficial skin necrosis); small incisions (4.5 cm); enhanced visibility; precise tissue handling with minimal scarring	High costs, small patient cohort, short follow-up period; limited long-term outcomes or comparison with conventional techniques
Lin et al., 2021 [17]	Scientific report	Head and neck: genioplasty	Craniofacial-plastic surgical robot (CPSR-I) system	Force feedback mechanism with automated drilling	Accurate osteotomy lines achieved high patient satisfaction without the need for additional surgeries	Heavy mechanical structure; insufficient navigation system for depth perception
Kanayama et al., 2021 [18]	Case series	Aesthetic: robotic recipient site preparation in hair transplantation	ARTAS Robotic System	Intelligent algorithms for hair identification; stereoscopic imaging	Efficient site creation with minimal complications; high patient and surgeon satisfaction ratings	It is limited to the frontal scalp and requires preoperative hair trimming; familiarity affects speed initially
Hwang et al., 2022 [19]	Case series	Chest reconstruction: RA latissimus dorsi muscle flap surgery for Poland Syndrome	da Vinci SP Surgical System	Flexible robotic arms; 3D imaging; gas insufflation	Mean operative time: 449 min; no perioperative complications; superior scar aesthetics; enhanced operator ergonomics	Longer setup time for robotic docking compared to manual methods; high cost of robotic equipment; steep learning curve
Wittesaele et al., 2022 [20]	Case series	Breast reconstruction: RA DIEP flap	da Vinci Xi Surgical System (Multiport)	Tremor elimination, motion scaling; 3D imaging	No flap losses or conversions; reduced fascial disruption; successful flap survival with minimal complications	Longer operative times due to the learning curve; limited patient selection; high equipment costs
Weinzierl et al., 2023 [21]	Case series	Microsurgery: lymphatic microsurgery, including VLNT and LVA	Symani Surgical System with conventional or 3D exoscope	Motion scaling; tremor elimination; 3D depth perception	Precise anastomoses (mean time: 22.6 min); 25.2% limb volume reduction at 3 months; no complications	High cost; steep learning curve; fixed robotic angles required longer incisions in deep spaces
Tsai et al., 2023 [22]	Cohort Study	Breast reconstruction: RA DIEP flap harvest	da Vinci Surgical System	Articulated robotic arms; high-definition 3D imaging	Reduced anterior rectus sheath incision length (RA 2.67 ± 1.13 cm vs. conventional 8.14 ± 1.69 cm); no flap loss; comparable pain scores; bilateral DIEP access achieved without robotic repositioning	RA procedure requires extra time (~100 min); costly disposables of approximately $3500; initial adaptation to post placement technique
Aman et al., 2024 [23]	Cohort study	Hand surgery: RA peripheral nerve surgery	Symani Surgical System	Tremor reduction; precise nerve coaptations	Coaptation time averaged 23 min; 100% patency achieved; some complications like hematoma were reported	High cost; technical logistics; inferior grip strength of instruments; steep learning curve
Dastagir et al., 2024 [24]	Case series	Hand surgery: microsurgical anastomosis	Symani Surgical System	NanoWrist instruments; motion scaling; tremor reduction	Patency rate of 100%; significant ergonomic benefits; anastomosis time reduced by 30% with experience	Steep learning curve; equipment positioning; high cost of devices and training
Tolksdorf et al., 2024 [25]	Case series	Craniofacial surgery: free flap surgery with robotic-assisted and conventional anastomosis	Symani Surgical System with Orbeye exoscope for 3D magnification	NanoWrists; 7 degrees of freedom; motion scaling; tremor filtering	Anastomosis times longer with robotic methods; flap survival comparable to manual methods; minor complications reported	Long learning curve; technical issues (arm collisions, software errors); inadequate grip strength for thin sutures
Gorji et al., 2024 [26]	Case series	Breast, trauma, and head and neck surgery: free flap reconstruction	Symani Surgical System and RoboticScope	Motion scaling, telemetric control, orbital view adjustment	95.7% flap survival; ischemia time 100.6 min; end-to-side anastomoses performed efficiently	High costs; increased surgical time; intraoperative ventilation issues; robot setup complexity
Könnecker et al., 2024 [27]	Case series	Limb reconstruction: free tissue transfer with microsurgical anastomosis for extremity reconstruction	Symani Surgical System	Motion scaling, tremor filtering, NanoWrists, ergonomic telemanipulators	Successful flap survival; mean anastomosis time 33.2 min; no vascular complications	Small sample size; lack of comparative manual data; challenging grip strength for delicate movements
Farr et al., 2024 [28]	Case series	Breast reconstruction: RANSM	da Vinci SP Robotic Surgical System	Flexible 3D camera, robotic arms with improved motion range	Median operative time was 277 min; learning curve improvement was observed with time; minor complications observed	Limited to a single surgeon; insufficient generalizability; technical ease dependent on breast size
Oh et al., 2024 [29]	Case series	Breast surgery: RA capsulectomy	da Vinci SP Robotic Surgical System	Curved scissors; bipolar forceps; 3D magnified imaging	Enhanced precision in capsulectomy; clear visualization in confined spaces	Longer operative times than conventional methods; high costs associated with robotic setup
Kim et al., 2024 [30]	Cohort study	Breast reconstruction: immediate breast reconstruction following mastectomy (implant-based and autologous DIEP flap reconstruction)	da Vinci SP Robotic Surgical System	Articulated robotic arms; motion scaling; tremor reduction	Fewer complications (skin necrosis 2% robotic vs. 8% conventional); improved sexual well-being scores	Longer operative times for robotic procedures; high costs; steep learning curve for surgeons
Wong et al., 2024 [31]	Case-control study	Breast reconstruction: free flap reconstruction after endoscopic or robotic mastectomy	da Vinci Xi Surgical System	Flexible robotic arms mimicking human wrist movements; 3D imaging and tremor reduction	Scar placement was aesthetically superior in the robotic group; 70.7% of scars were hidden laterally compared to 70.7% in the conventional group	Aesthetic revision rates similar to conventional mastectomy; no direct aesthetic advantage with da Vinci Xi apart from scar position
von Reibnitz et al., 2024 [32]	Case series	Microsurgery: RA lymphatic reconstruction with LTT and/or LVA or LLA	Symani Surgical System	Motion scaling; tremor reduction; 7 degrees of freedom	Limb volume reduction of 1-10% (80 to 1250 mL); wound healing complications in 6.4% of cases; no need for surgical assistant	Lack of direct comparison with manual methods; inability to monitor long-term flap survival; single-center study
Jung et al., 2024 [33]	Cohort study	Breast reconstruction: RANSM with immediate breast reconstruction	da Vinci Xi Surgical System	Not specified	Significant improvements in operation times after 21 cases; mean docking time dropped from 8.2 to 5 min; minimal complications	Single-surgeon study; technical difficulties with the multiport systems, such as arm collisions and camera blind spots; limited tactile sensation was reported compared to conventional NSM

RA = robot assisted, NSM = nipple-sparing mastectomy, RANSM = robot-assisted nipple-sparing mastectomy, DIEP = deep inferior epigastric perforator, VLNT = vascularized lymph node transfer, LVA = lymphaticovenous anastomosis, LTT = lymphatic tissue transfer, LLA = lympholymphatic anastomoses.

**Table 2:** Advantages and disadvantages of different robotic system.

Robotic System	Advantages	Disadvantages
Da Vinci Surgical System	High precision Enhanced 3D visualization Motion scaling and tremor elimination Articulated robotic arms mimicking human wrist movements Wide range of surgical applications (breast reconstruction, head and neck surgery, microsurgery)	High cost Lack of tactical feedback Steep learning curve especially for complex procedures Longer setup and docking times
Symani Surgical System	Microsurgical procedures Tremor filtration and reduction Compact design Motion scaling up to 20× NanoWrists with 7 degrees of freedom Superior precision for supermicrosurgery	Limited to niche microsurgical application High initial cost Requires significant operating room space
ARTAS Robotic System	Intelligent algorithms for hair graft placement (algorithm-driven follicle extraction) Minimal trauma to surrounding tissues High patient and surgeon satisfaction	Limited to hair restoration procedures Requires preoperative hair trimming High cost and training requirements
Craniofacial Plastic Surgical Robot (CPSR-I)	Force feedback mechanism Accurate osteotomy for craniofacial surgery Reduced need for revision surgery	Heavy and bulky mechanical structure Insufficient navigation system for depth perception Limited to craniofacial application Compatibility issues with imaging systems

## Data Availability

The authors confirm that the data supporting this study’s findings are available within the article.

## Abbreviations

The following abbreviations are used in this manuscript:

RA	Robot assisted
NSM	Nipple-sparing mastectomy
RANSM	Robot-assisted nipple-sparing mastectomy
DIEP	Deep inferior epigastric perforator
VLNT	Vascularized lymph node transfer
LVA	Lymphaticovenous anastomosis
LTT	Lymphatic tissue transfer
LLA	Lympholymphatic anastomoses
